# Examining the impact of zinc on horizontal gene transfer in Enterobacterales

**DOI:** 10.1038/s41598-022-23690-z

**Published:** 2022-11-28

**Authors:** Daniel Ekhlas, Arturo B. Soro, Finola C. Leonard, Edgar G. Manzanilla, Catherine M. Burgess

**Affiliations:** 1 Department of Food Safety, Teagasc Food Research Centre, Ashtown, Dublin, Ireland; 2 School of Veterinary Medicine, University College Dublin, Dublin, Ireland; 3 Department of Food Chemistry & Technology, Teagasc Food Research Centre, Ashtown, Dublin, Ireland; 4 Pig Development Department, Teagasc Moorepark, Fermoy, Co. Cork Ireland

**Keywords:** Antimicrobial resistance, Microbiology, Antimicrobials

## Abstract

Antimicrobial resistance is one of the main international health concerns for humans, animals, and the environment, and substantial efforts have focused on reducing its development and spread. While there is evidence for correlations between antimicrobial usage and antimicrobial resistance development, specific information on the effect of heavy metal/antimicrobial usage on bacterial conjugation is more limited. The aim of this study was to investigate the effects of zinc and antimicrobials in different concentrations on horizontal gene transfer of an ampicillin resistance gene, using a multi-drug resistant *Escherichia coli* donor strain and three different *Salmonella enterica* serovars as recipient strains. Differences in conjugation frequencies for the different *Salmonella* recipients were observed, independent of the presence of zinc or the antimicrobials. Selective pressure on the recipient strains, in the form of ampicillin, resulted in a decrease in conjugation frequencies, while, the presence of rifampicin resulted in increases. Zinc exposure affected conjugation frequencies of only one of the three recipient strains, thus the effect of zinc on conjugation frequencies seemed to be concentration and strain dependent. Furthermore, differences in growth rates due to plasmid carriage were observed for one of the *Salmonella* strains.

## Introduction

Bacterial conjugation plays an important role in the persistence and adaption of bacteria to different environments^1^. Transferred mobile genetic elements, such as conjugative plasmids, can harbour various traits, including virulence factors and antimicrobial resistance genes (ARGs), which can increase the overall fitness of bacteria^2^. This can be problematic in clinical and agricultural settings where human and animal health is reliant on the effectiveness of antimicrobials^3^. Moreover, in these settings heavy metals and antimicrobials are used for prevention and treatment of infectious disease. For example, therapeutic levels of zinc oxide (2500–3000 ppm) are frequently administered during weaning on commercial pig farms to prevent infection with enteric pathogens. Such usage was banned by the European Union from June 2022^4–6^. In the last decade, increasing evidence indicates that the use of antimicrobials and heavy metals can be associated with the development and spread of antimicrobial resistance (AMR)^2,7^. Although the effects of antimicrobials and heavy metals on bacterial conjugation are not fully understood, links between antimicrobial and heavy metal use with AMR transfer pose a high risk in settings where their use is common, such as in primary production. Inappropriate excessive use of antimicrobials and heavy metals could promote AMR transfer, thus contributing to the development of multi-drug resistant pathogens, which limits treatment options, increases the associated economic burden of treatment costs, and is potentially a hazard to public health^8^.

A study by Beaber et al.^9^ reported evidence that the bacterial SOS response promotes horizontal gene transfer (HGT). Since then, several studies have focused on the associations between bacterial conjugation and bacterial stress, particularly those induced by exposure to antibiotics and heavy metals, with varying results^10–13^. There is a lack of clarity about the factors influencing the rate of HGT between strains and species, although some evidence suggests that sub-inhibitory concentrations of antimicrobials can promote HGT. Treatment of animals in primary production with antimicrobials, some of which are often not fully absorbed due to their low bioavailability, can result in antimicrobial residues in livestock manure which may subsequently be used as fertiliser. This may lead to the further distribution of sub-inhibitory concentrations of antimicrobials into soil or aquatic systems in the form of water runoff from agricultural land^14,15^. There are several different routes connecting animals, humans, and the environment, which may not only allow the distribution of antimicrobial residues but also the associated development and spread of AMR in potential pathogens^7^. It is therefore necessary to gain a greater understanding on how antimicrobial residues may affect the spread and development of AMR and how to prevent it, as part of a multi-sectorial One Health approach to tackle AMR^16^.

The aim of this study was to elucidate the effect of sub-inhibitory concentrations of zinc, in comparison to selected antimicrobials, on HGT in *E. coli*, using multiple *Salmonella* recipient strains. Furthermore, conjugation transfer was monitored temporally to assess how sub-inhibitory concentrations of zinc affect HGT at different stages of bacterial growth. Additionally, this study investigated the effects of plasmid carriage on growth as an indicator of bacterial fitness.

## Methodology

### Bacterial strains

One *E. coli* donor and three *Salmonella* recipient strains were selected from the culture collection in the Food Safety Department at Teagasc Food Research Centre Ashtown in Dublin, based on preliminary experiments. An extended-spectrum beta-lactamase (ESBL) carrying *Escherichia coli* isolate (serotype: O9:H17) from avian faeces was selected as a donor strain. Three *Salmonella enterica* strains, *S.* Infantis (origin: pig faeces; serotype: ?:r:1,5), *S.* London (origin: pig feed bin; serotype: 3,10:l,v:1,6), and *S.* Enteriditis (origin: poultry; serotype: 9:g,m:-) were selected as recipient strains.

### Preparation of rif^R^ recipient mutants

To aid enumeration, rifampicin-resistant (rif^R^) mutants of the wild type (WT) recipient strains were raised based on the method described by Blackburn and Davies^17^. Briefly, overnight cultures (ONCs) of the recipient strains were prepared, diluted to a concentration of 7 log_10_ CFU/mL and transferred into three Eppendorf tubes per WT recipient strain, containing 1 mL Tryptone Soya Broth (TSB, Oxoid, United Kingdom) with an initial rifampicin (reagent grade 95%, Sigma-Aldrich, Ireland) concentration of 12.5 µg/mL, which were incubated for 24 h at 37 °C without shaking. Tubes that showed growth were selected and suspensions diluted as for the ONCs. Dilutions were transferred again into fresh TSB with 25 µg/mL rifampicin, incubated, and repeated with increasing rifampicin concentrations until growth in TSB with 200 µg/mL rifampicin was observed. Dilutions of this suspension were plated on Tryptone Soya Agar (TSA, Oxoid, United Kingdom), containing 200 µg/mL rifampicin and incubated for 24 h at 37 °C. For each recipient strain rif^R^ mutants were isolated and stored on cryogenic beads (Technical Service Consultant Ltd., United Kingdom) at − 20 °C.

The mutants were analysed for the stability of the mutation which conferred rifampicin resistance. ONCs of the mutants were prepared and sub-cultured twice a day in TSB for 7 days and incubated at 37 °C. After 7 days, dilutions of the bacterial suspensions were plated on TSA containing 200 µg/mL rifampicin to confirm they retained rifampicin resistance. The antimicrobial susceptibility profiles and minimal inhibitory concentrations (MICs) were determined for the rif^R^ mutants as described below.

### Antimicrobial resistance profiling

Donor and recipient strains were analysed for antimicrobial susceptibility using the Kirby–Bauer disc diffusion susceptibility test and MIC testing.

For the disc diffusion test, strains were streaked on TSA and incubated for 24 h at 37 °C. Subsequently, ONCs were prepared by transferring one colony of each strain into 10 mL TSB and incubated for 16 h at 37 °C without shaking. The ONCs were diluted 1:100 using Maximum Recovery Diluent (MRD, Oxoid, United Kingdom) to obtain a concentration of 7 log_10_ CFU/mL, which approximately matched the 0.5 McFarland turbidity standard. Bacterial suspensions were spread with cotton swabs on Mueller–Hinton Agar (MHA, Oxoid, United Kingdom) in duplicate. Subsequently, eight different antibiotic containing discs [trimethoprim (5 µg), nalidixic acid (30 µg), tetracycline (30 µg), meropenem (10 µg), cefotaxime (5 µg), ciprofloxacin (5 µg), streptomycin (10 µg), and chloramphenicol (30 µg), Oxoid, United Kingdom] were applied on the inoculated MHA plates and were incubated for 24 h at 37 °C. The reference *E. coli* strain ATCC 25922 was used as a control. After incubation the diameters of the inhibition zones were measured and interpreted according to breakpoint standards published by the European Committee on Antimicrobial Susceptibility Testing^18^ and the Clinical and Laboratory Standards Institute^19^.

For determination of the MIC, ONCs for all strains were prepared and diluted to obtain suspensions with a concentration of approximately 3 log_10_ CFU/mL. For each strain 100 µL of the bacterial suspension was transferred into 96-well plates and mixed with 100 µL Mueller–Hinton Broth (MHB, Oxoid, United Kingdom), containing decreasing concentrations of the test antimicrobial, which was in this case rifampicin, ampicillin sodium salt (Sigma-Aldrich, Ireland), or zinc nitrate hexahydrate (reagent grade 98%, Sigma Aldrich, Ireland). Prior to their use, test components were dissolved in sterile deionised water and filtered through aseptic 0.22 µm syringe filters (GVS, USA). Antimicrobial concentrations were two-fold diluted resulting in test ranges from 0.5 to 12 mM of zinc nitrate for all strains; 0.125–16 µg/mL and 16–512 µg/mL of ampicillin for recipients, donor and transconjugants respectively; 0.25–16 µg/mL and 25–800 µg/mL of rifampicin for donor, recipients and transconjugants respectively. The strains were tested in triplicate in two independent experimental runs for each test component. The MIC assays were incubated for 24 h at 37 °C and MICs, defined as the lowest concentration at which no growth was observed, were determined via visual assessment.

### Conjugation assay

ONCs of the donor strain and rif^R^ mutant strains were prepared and diluted to a concentration of approx. 6 log_10_ CFU/mL. One mL of each dilution of the donor and individual recipients were transferred into tubes with 8 mL TSB containing different concentrations of either Zn(NO_3_)_2_ (0 mM, 0.25 mM, 1 mM), rifampicin (0 µg/mL, 2 µg/mL, 8 µg/mL), or ampicillin (0 µg/mL, 0.25 µg/mL, 0.5 µg/mL). One ONC served as an inoculum for two technical duplicates. As controls, pure cultures were prepared by adding 1 mL of donor or recipient dilutions to 9 mL TSB. All mixtures were vortexed and incubated for 24 h at 37 °C without shaking. After incubation, bacterial suspensions were serial diluted and 100 µL plated on Xylose Lysine Deoxycholate (XLD, Oxoid, United Kingdom) Agar, containing 100 µg/mL rifampicin and ampicillin, respectively.

Control cultures were also serial diluted and plated on either Tryptone Bile X-Glucuronide (TBX, Merck KGaA, Germany) Agar for the donor strain or XLD Agar, containing 100 µg/mL rifampicin for recipient strains. All XLD plates were incubated for 24 h at 37 °C, while TBX plates were incubated for 24 h at 44 °C. After incubation, plate counts for transconjugants and controls were obtained. Conjugation frequencies were calculated by dividing transconjugant numbers by recipient numbers (see Eq. 1).

1 $$Conjugation \, frequency= {log}_{10} \left(\frac{Transconjugant \,numbers [\frac{CFU}{mL}]}{Recipient\, numbers [\frac{CFU}{mL}]}\right)$$

Each conjugation experiment was repeated three times. Transconjugant colonies exposed to 0 mM and 1 mM Zn(NO_3_)_2_ were transferred into cryogenic tubes and stored at − 80 °C for subsequent analysis via whole genome sequencing (WGS). Additionally, transconjugants were analysed using the previously described Kirby-Bauer disc diffusion test and the MIC assay.

### Impact of time on conjugation

The effect of time on conjugation in the presence of Zn(NO_3_)_2_ exposure was assessed with *S.* London as the recipient. *S.* London and the donor ESBL *E. coli* strain were mixed together in the presence of different concentrations of Zn(NO_3_)_2_, using a similar protocol as for the previous conjugation assay. After inoculation of both strains, a sample was taken and plated immediately (t0). The tubes were incubated for 24 h at 37 °C without shaking. Direct samples were then taken hourly for the first 8 h (t1-t8), and plated on double-selective XLD agar, containing 100 µg/mL rifampicin and ampicillin, respectively (for transconjugant numbers), mono-selective XLD agar, containing 100 µg/mL rifampicin (for recipient and transconjugant numbers), and non-selective TBX agar (for donor numbers). A final sample was taken after 24 h (t24). The experiment was performed three times, running the conjugation set-up in duplicate per test concentration and plating in duplicate, respectively.

### Comparison of bacterial growth

ONCs of donor, rif^R^ recipient strains, and stored transconjugants were prepared and diluted with MRD to give an approximate concentration of 4 log_10_ CFU/mL. From the dilutions, 100 µL bacterial cell suspension was transferred to 96-well plates and mixed with 100 µL TSB. For the negative control 100 µL sterile TSB and 100 µL MRD were mixed together. Bacterial growth at 37 °C with continuous shaking was monitored for 24 h at an optical density of 595 nm (OD_595 nm_) using a Multiskan™ FC microplate photometer (ThermoFisher Scientific, UK) and the SkanIt Software (v4.1.0.43, ThermoFisher Scientific). The optical density was measured every 30 min. The Gompertz model was fitted to data points via the DMFit 3.5 Microsoft Excel add-in to obtain fitted growth curves of  the donor, rif^R^ recipient strains, and transconjugants. Furthermore, fitted growth curves were evaluated qualitatively using the regression coefficient R^2^ as a measures of goodness-of-fit. Growth model fitting and statistical evaluation of fitted models were in accordance with the equations used in a study by Soro et al.^20^.

### Whole genome sequencing and analysis

DNA of the stored transconjugants from the conjugation assay, rif^R^ recipient strains, and the donor strain was extracted using the DNeasy® Blood & Tissue kit from Qiagen (Germany, Cat. No. 69506). DNA extracts were checked for their purity using Nanodrop™ 1000 Spectrophotometer and were quantified using Qubit 4.0 Fluorometer (Invitrogen, ThermoFisher Scientific, United Kingdom). Subsequently, DNA extracts were sent to Novogene Co. Ltd. (United Kingdom) for WGS on the Illumina® NovaSeq 6000 platform. Obtained raw sequences were cleaned in a 3-step filtering process in which: (1) adapters were removed, (2) reads containing more than 10% undetermined bases [N > 10%] were removed, and (3) low quality reads for which 50% of total bases had a Qscore below or equal to 5 (Qscore ≤ 5) were removed.

Cleaned raw reads were quality assessed using FastQC (v0.11.8) and MultiQC (v1.9)^21,22^. Strains were identified using Kraken 2 (v2.0.7 beta), using the standard Kraken 2 (last update: 2020-Apr-11) database^23^. Additionally, serotypes were confirmed for the *E. coli* donor strain and for the *S. enterica* recipients, using the online tool SerotypeFinder 2.0 from the Center for Genomic Epidemiology and the online tool SISTR^24,25^. After strain confirmation with the cleaned raw reads, reads were assembled to contigs and scaffolds using SPAdes (v3.13.0) and its –careful option^26^. Assembly quality of scaffolds was assessed via QUAST (v5.1.0) in combination with MultiQC^22,27^. Genomic scaffolds were then used for prokaryotic genome annotation via Prokka (v1.14.6) and were aligned via ABRricate (v0.8; https://github.com/tseemann/abricate) against the ResFinder (last update: 2020-Apr-16) and the PlasmidFinder (last update: 2020-Apr-16) databases, to screen for antimicrobial resistance genes and plasmids respectively^28–30^. Identified ARGs and plasmids were compared between donor, recipient and transconjugant strains to determine which elements were transferred during the conjugation assay.

Subsequently, cleaned raw reads were again assembled using plasmidSPAdes (v3.13.0), in an attempt to reconstruct the transferred plasmid that harboured ARGs conferring the observed ampicillin resistance in donor and transconjugants^31^. Assembled plasmid scaffolds were once more checked using QUAST (v5.1.0) and MultiQC^22,27^. Plasmid scaffolds were further analysed using Prokka (v1.14.6) and ABRicate (v0.8; https://github.com/tseemann/abricate) as mentioned above^28–30^.

### Visualization and statistical methods

All results of the conjugation studies were analysed using R (v.4.0.2)^32^. Visualization of results was done using the “ggplot2” package in R^33^. The impact of Zn(NO_3_)_2_, rifampicin, and ampicillin on conjugation frequencies and transconjugant numbers in the conjugation assay was assessed using the Student’s *T*-Test (R function “*t*_test” in R package “rstatix”)^34^. Resulting p-values were adjusted via the Bonferroni correction and significance was set to α = 0.05. To allow statistical comparison when zero transconjugants were observed within the conjugation studies, the lower limit of detection was calculated as 1 CFU/mL, similar to Crane et al.^11^. Furthermore, the calculation of conjugation frequencies was omitted for data points with no detected transconjugants. Visualization of fitted growth curves and was done using GraphPad Prism 8 (v8.4.2) for Windows (GraphPad software Inc, San Diego, CA, USA). Growth curve parameters were compared using a one-way ANOVA together with the Tukey post-hoc test (α = 0.05) via Minitab 17 (v17.1.0). The results of the bioinformatics analysis were used in combination with ApE—A plasmid Editor Software tool (http://biologylabs.utah.edu/jorgensen/wayned/ape/) to reconstruct and visualize the primary transferred plasmid in the conjugation assay. Graphs were combined to figures and their quality optimised by using the vector graphics editor Affinity Designer (v1.8.5.703, Serif).

## Results

### Antimicrobial resistance profiling

The MICs for ampicillin, rifampicin and Zn(NO_3_)_2_ for the donor, recipients and transconjugants are shown in Table 1. Furthermore, the donor strain was resistant to tetracycline, cefotaxime, streptomycin, and had an intermediate resistance to ciprofloxacin. The rif^R^ recipient strains were resistant to rifampicin, while the transconjugants were resistant to rifampicin and ampicillin only, indicating that of the antimicrobials tested only resistance to ampicillin was transferred.

**Table 1:** Antimicrobial resistance profiles identified via MIC testing of donor, recipients, and transconjugants.

Strains	Ampicillin [µg/mL]	Rifampicin [µg/mL]	Zn(NO_3_)_2_ [mM]
**Donor**			
ESBL *E. coli*	512	8	1
**WT Recipients**			
*S. Infantis*	0.5	16	1
*S. London*	0.5	16	1
*S. Enteriditis*	0.5	8	1
**Raised rif**^***R***^** recipients**			
*S. Infantis rif*^*R*^* C5*	0.5	> 800	1
*S. London rif*^*R*^* C8*	0.5	> 800	1
*S. Enteriditis rif*^*R*^* C2*	0.5	> 800	1
***S. Infantis Transconjugants***			
Conjugation with 0 mM Zn(NO_3_)_2_	256	> 800	1
Conjugation with 0.25 mM Zn(NO_3_)_2_	256	> 800	1
Conjugation with 1 mM Zn(NO_3_)_2_	256	> 800	1
***S. London Transconjugants***			
Conjugation with 0 mM Zn(NO_3_)_2_	512	> 800	1
Conjugation with 0.25 mM Zn(NO_3_)_2_	512	> 800	1
Conjugation with 1 mM Zn(NO_3_)_2_	512	> 800	1
***S. Enteriditis Transconjugants***			
Conjugation with 0 mM Zn(NO_3_)_2_	128	> 800	1
Conjugation with 0.25 mM Zn(NO_3_)_2_	128	> 800	1
Conjugation with 1 mM Zn(NO_3_)_2_	128	> 800	1

### Conjugation assay

**Figure 1:**
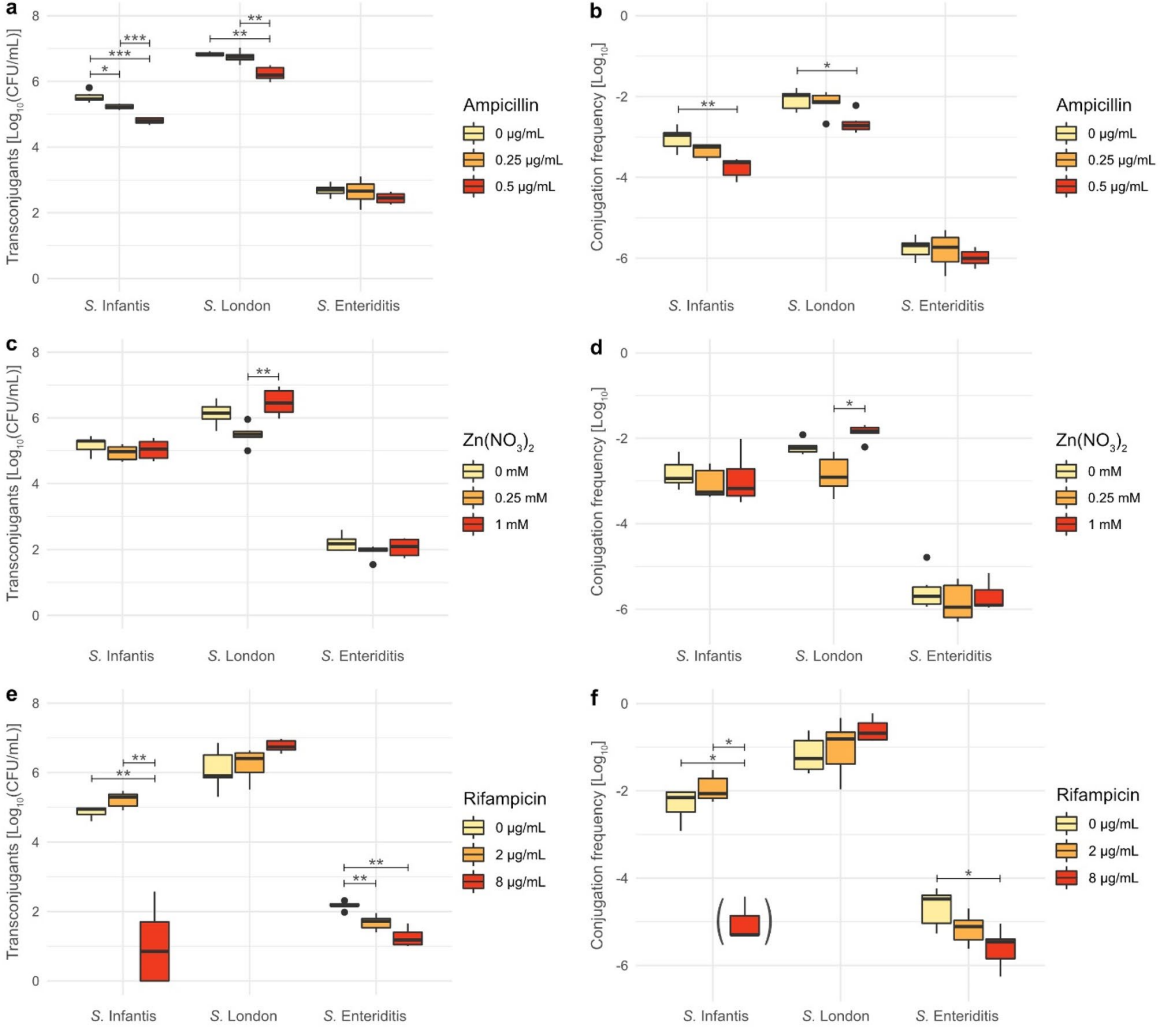
Transconjugant numbers and calculated conjugation frequencies, observed at different concentrations of ampicillin (**a**,**b**), Zn(NO_3_)_2_ (**c**,**d**), and rifampicin (**e**,**f**). Pairwise comparison of transconjugant numbers and conjugation frequencies at different concentrations was perform via the Student’s *T*-test. *P*-values were adjusted using the Bonferroni correction. Significance levels: *p < 0.05, **p < 0.01, ***p < 0.001. Boxplot in brackets (**f**) indicates that the calculation of conjugation frequencies for some data points was omitted due to no observed transconjugants.

In the conjugation studies, although all three rif^R^ recipient strains belonged to the same species, differing transconjugant numbers were observed for the different serovars, independent of the exposure to potential selective pressure (Fig. 1 a,c,e). Of these serovars *S.* Enteriditis showed the lowest transconjugant numbers, while *S.* London showed the highest (Fig. 1a,c,e). *S.* Infantis showed similar transconjugant numbers to *S.* London. Conjugation frequencies correlated with observed transconjugant numbers (Fig. 1b,d,f).

In the presence of ampicillin, transconjugant numbers and conjugation frequencies for *S.* Infantis and *S.* London decreased (*p* < 0.01) with increasing ampicillin concentration, in contrast to *S.* Enteriditis which was unaffected (Fig. 1a,b). In the presence of Zn(NO_3_)_2_ no effect on either transconjugant numbers or conjugation frequencies was observed for all three recipient strains, except for the conjugation frequency for *S.* London which was reduced at lower concentrations of Zn(NO_3_)_2_ (0.25 mM, *p* < 0.05). This effect was also observed for transconjugant numbers, but without reaching significance. Furthermore, a slight increase of transconjugant numbers and conjugation frequencies were observed for the highest concentration of Zn(NO_3_)_2_ (1 mM) in comparison to no exposure to Zn(NO_3_)_2_, although this increase was not significant. The presence of rifampicin increased conjugation frequencies at 2 µg/mL, but decreased conjugation frequencies (*p* < 0.05) at higher concentrations (8 µg/mL) for *S.* Infantis and *S.* Enteriditis. Interestingly, *S.* London was affected differently by higher concentrations of rifampicin than the other two recipient strains, showing non significant increases in transconjugant numbers and conjugation frequencies (*p* > 0.05).

Transconjugants from one run were investigated by WGS analysis, as well as by antimicrobial susceptibility testing. AMR profiles of these transconjugants showed no changes in resistance to rifampicin and Zn(NO_3_)_2_ when compared to the original rif^R^ recipient strain. However, all transconjugants showed increased resistance to ampicillin (Table 1). Furthermore, the MICs for ampicillin were different for each transconjugant strain, with *S.* Enteriditis showing the lowest ampicillin MIC (128 µg/mL), while *S.* Infantis, which had the highest conjugation frequency of all three recipient strains, showed the highest MIC for ampicillin (512 µg/mL) (Table 1; Fig. 1a,c,e).

### Impact of time on conjugation

**Figure 2:**
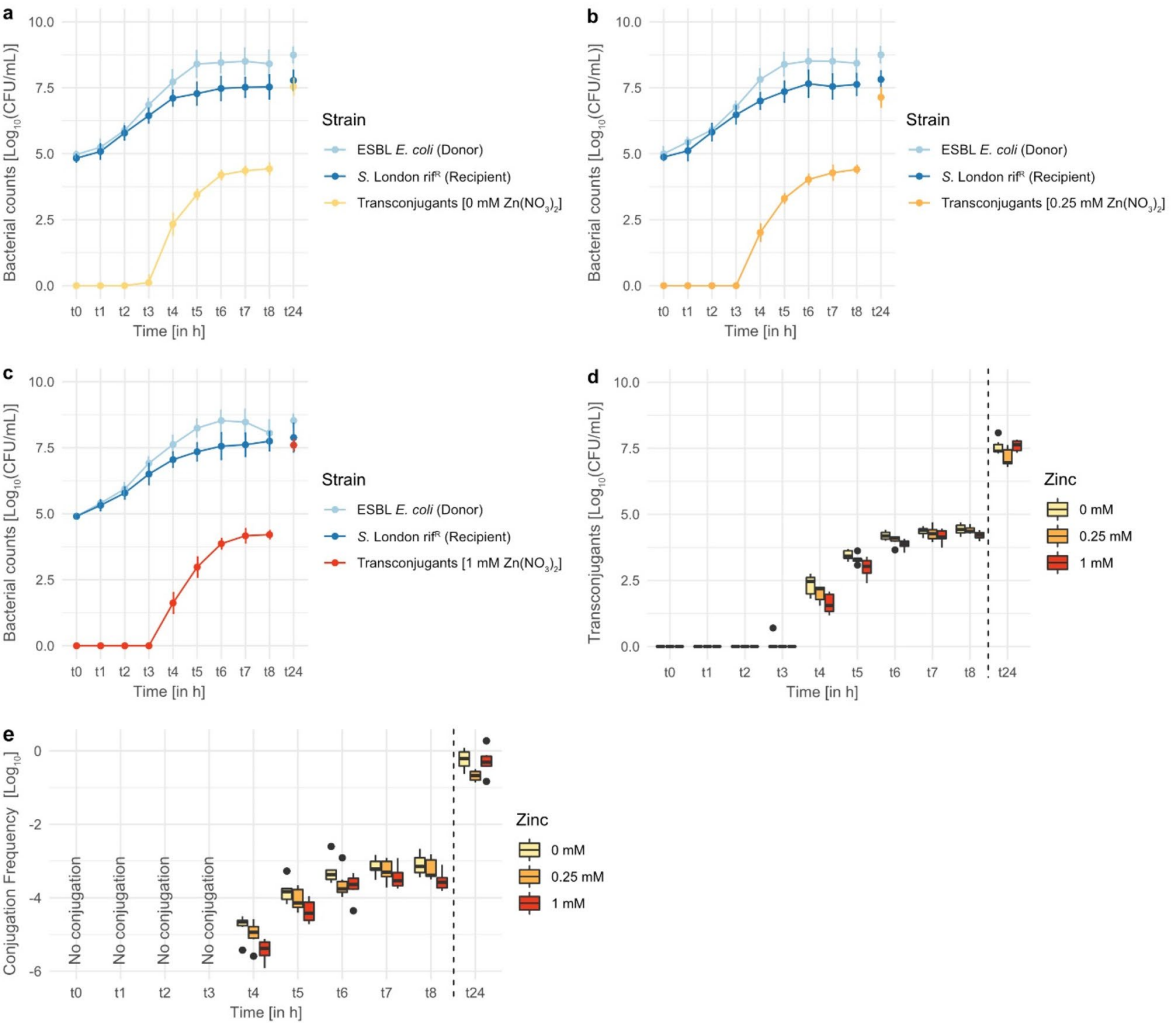
Transconjugant, donor, and recipient numbers during exposure to (**a**) 0 mM, (**b**) 0.25 mM, and (**c**) 1 mM of Zn(NO_3_)_2_, from inoculation [t0] measured hourly for 8 h [t8] and additionally measured after 24 h [t24]. (**d**) Transconjugant numbers and (**e**) conjugation frequencies at different concentrations of Zn(NO_3_)_2_. Pairwise comparison of transconjugant numbers and conjugation frequencies at different concentrations was performed via Student’s *T*-test. *P*-values were adjusted using the Bonferroni correction. No significant differences were observed.

To obtain a deeper understanding of the impact of Zn(NO_3_)_2_ on conjugation and its timing, another conjugation assay was set up with hourly sampling for 8 h and a final sampling 24 h post-inoculation. The rif^R^ recipient strain *S.* London was used, as this recipient was the only one showing differences in conjugation frequencies during exposure to Zn(NO_3_)_2_ (Fig. 1d). The observed donor and rif^R^ recipient numbers were the same for each time sample (t0–t24), independent of the concentration of Zn(NO_3_)_2_ (Fig. 2a–c).

The first transconjugants were obtained 4 h post-inoculation (t4), followed by an exponential increase of transconjugant numbers which slowly declined 5–6 h post-inoculation, similar to a bacterial growth curve. Higher transconjugant numbers and conjugation frequencies were observed in the absence of Zn(NO_3_)_2_ exposure until 8 h (t8) post-inoculation, but were not significantly different (Fig. 2d,e). After 24 h the same pattern as the original conjugation assay for *S.* London were seen, in which transconjugant numbers and conjugation frequencies were negatively affected by lower concentrations (0.25 mM) of Zn(NO_3_)_2_, but not by higher concentrations (1 mM). In contrast to the original conjugation assay, these differences were not significant (*p* > 0.05), which could be due to experimental conditions and biological variability.

### Comparison of bacterial growth

**Figure 3:**
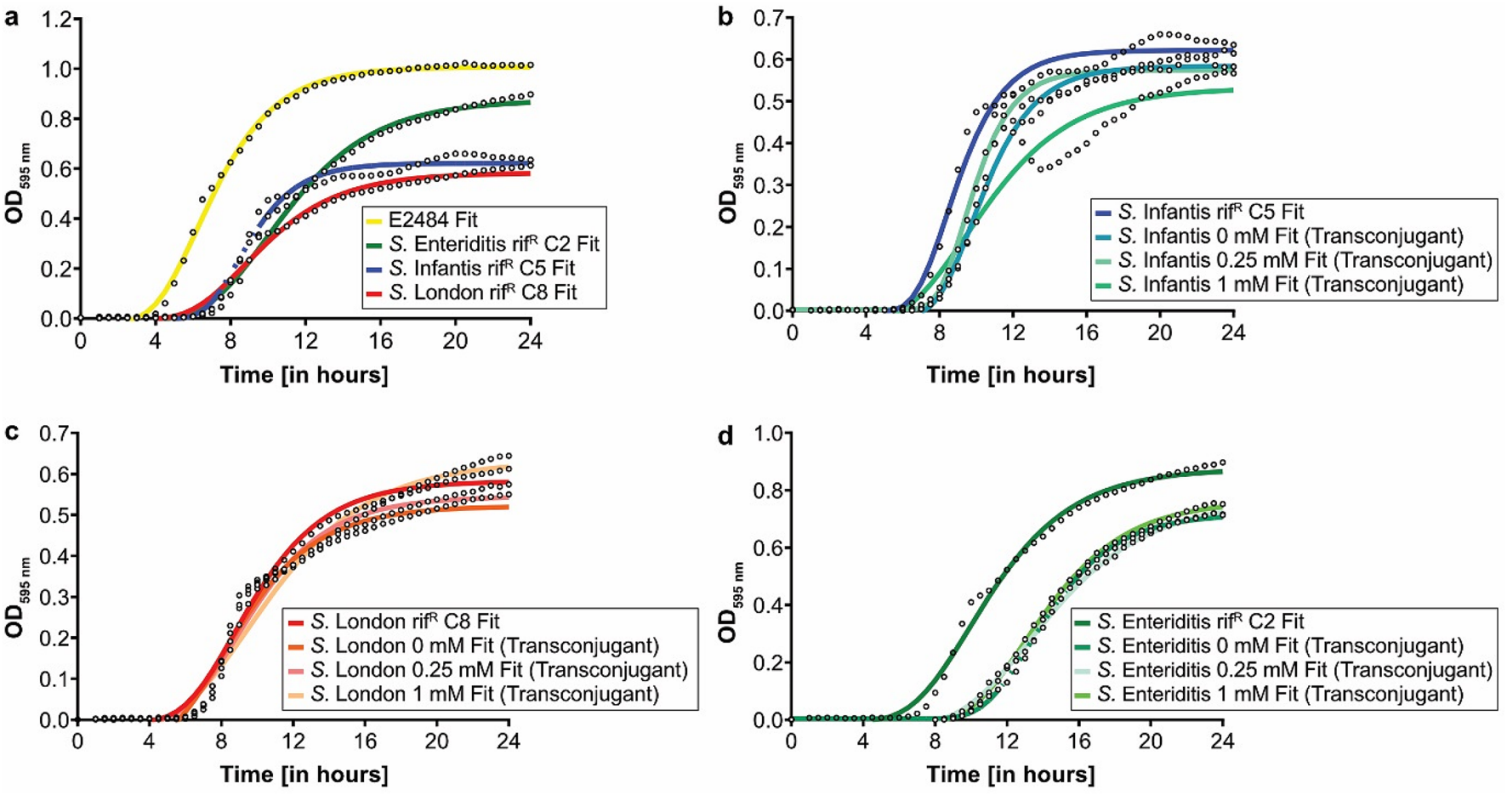
Gompertz-fitted growth curves of donor, recipients, and transconjugants with (**a**) comparisons of donor and recipient strain, (**b**–**d**) comparisons of transconjugants with their parent recipient strain.

To assess differences between the strains used, as well as to assess if the fitness of transconjugants was impacted by the transferred plasmid, the growth of donor, recipient, and transconjugant strains were compared and growth data was fitted using the Gompertz equation. Comparison of the donor and recipient strains showed that all four strains grew similarly to each other. However, the maximum growth rate (µ_max_) of the donor was higher than the *S.* London recipient strain (Table 2). Furthermore, the lag phase (λ) of the donor strain was shorter compared to all recipient strains. The final concentration (Y_End_) of donor and the *S.* Enteriditis recipient strain were both similar to each other and higher compared to the other two recipient strains. Fitted growth curves of all strains can be seen in Fig. 3.

**Table 2:** Growth curve parameters of fitted Gompertz models of donor, recipients, and transconjugants, with µ_max_ (maximum growth rate), λ (lag phase), Y_0_ (initial concentration), Y_End_ (final concentration), and the regression coefficient R^2^ as a measure of goodness-of-fit. Parameters were compared using a one-way ANOVA and the Tukey post-hoc test with α = 0.05. Groups that differ significantly for each fitted parameter are denoted by different superscripts.

Strains	µ_max_	λ	Y_0_	Y_End_	R^2^
**Donor**					
ESBL* E. coli*	0.167 ± 0.017^A^	3.801 ± 0.405^D^	− 0.040 ± 0.044^C^	1.013 ± 0.144^A^	0.994 ± 0.006
**Raised rif**^***R***^** recipients**					
*S. Infantis rif*^*R*^* C5*	0.140 ± 0.034^ABC^	6.690 ± 0.261^BC^	− 0.002 ± 0.005^ABC^	0.625 ± 0.050^C^	0.979 ± 0.012
*S. London rif*^*R*^* C8*	0.082 ± 0.010^CD^	6.024 ± 0.321^C^	− 0.006 ± 0.006^ABC^	0.586 ± 0.113^C^	0.988 ± 0.005
*S. Enteriditis rif*^*R*^* C2*	0.110 ± 0.014^ABCD^	6.895 ± 0.208^BC^	− 0.005 ± 0.009^ABC^	0.897 ± 0.221^AB^	0.994 ± 0.002
**S. *Infantis Transconjugants***					
Conjugation with 0 mM Zn(NO_3_)_2_	0.155 ± 0.061^AB^	8.318 ± 0.977^B^	− 0.006 ± 0.027^ABC^	0.581 ± 0.084^C^	0.962 ± 0.032
Conjugation with 0.25 mM Zn(NO_3_)_2_	0.166 ± 0.073^A^	7.936 ± 0.462^B^	0.008 ± 0.013^A^	0.581 ± 0.082^C^	0.976 ± 0.012
Conjugation with 1 mM Zn(NO_3_)_2_	0.106 ± 0.071^BCD^	6.590 ± 2.008^BC^	− 0.025 ± 0.030^ABC^	0.590 ± 0.194^C^	0.913 ± 0.068
**S. *London Transconjugants***					
Conjugation with 0 mM Zn(NO_3_)_2_	0.080 ± 0.004^D^	5.641 ± 0.549^CD^	− 0.037 ± 0.029^BC^	0.530 ± 0.168^C^	0.978 ± 0.010
Conjugation with 0.25 mM Zn(NO_3_)_2_	0.082 ± 0.007^CD^	5.975 ± 0.520^C^	− 0.024 ± 0.029^ABC^	0.554 ± 0.216^C^	0.980 ± 0.011
Conjugation with 1 mM Zn(NO_3_)_2_	0.071 ± 0.007^D^	5.8 56 ± 0.537^C^	− 0.027 ± 0.027^ABC^	0.643 ± 0.232^C^	0.983 ± 0.008
**S. *Enteriditis Transconjugants***					
Conjugation with 0 mM Zn(NO_3_)_2_	0.114 ± 0.026^ABCD^	11.356 ± 1.486^A^	0.011 ± 0.006^A^	0.727 ± 0.115^BC^	0.996 ± 0.002
Conjugation with 0.25 mM Zn(NO_3_)_2_	0.114 ± 0.024^ABCD^	11.045 ± 2.474^A^	0.007 ± 0.021^A^	0.728 ± 0.100^BC^	0.993 ± 0.006
Conjugation with 1 mM Zn(NO_3_)_2_	0.124 ± 0.025^ABCD^	10.921 ± 1.699^A^	0.000 ± 0.037^AB^	0.752 ± 0.117^BC^	0.993 ± 0.008

When comparing transconjugant strains with their parent recipient strain, differences can be seen for the lag phase of *S.* Enteriditis, with the lag phase of the *S.* Enteriditis transconjugants longer than their parent recipient strain. No differences in the maximum growth rates were observed between the transconjugants and their corresponding recipient strains but the maximum growth rate and lag phase were strain dependent.

Evaluation of each fitted growth curve with the regression coefficient R^2^ as a measure of goodness-of-fit showed that each fitted curve represented the measured growth data well (Table 2).

### Whole genome sequencing and analysis

**Figure 4:**
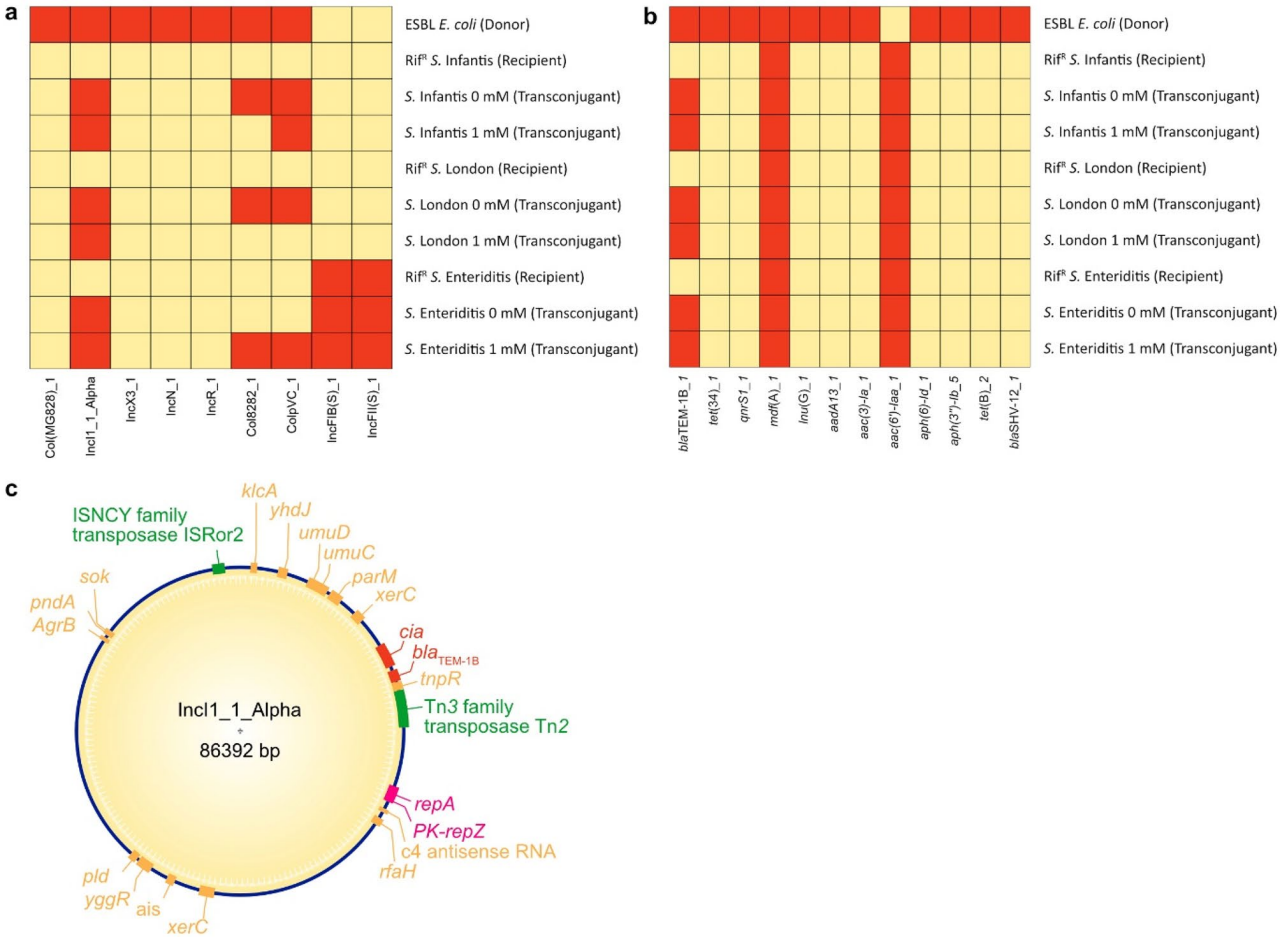
Results of the WGS analysis via ABRicate, represented in the form of heatmaps, showing (**a**) comparison of observed plasmids and (**b**) antimicrobial resistance genes (highlighted in red) between donor, rif^R^ recipients, and transconjugants of the conjugation assay with Zn(NO_3_)_2_. (**c**) Reconstruction of the transferred plasmid from donor to recipients (transposases highlighted in green; genes involved in plasmid replication highlighted in purple; genes involved in AMR highlighted in red).

The purpose of the WGS analysis was to determine which plasmid and ARGs were transferred from the donor to the recipient strains. Resistance profiles were analysed using ResFinder (Fig. 4a), and plasmids were analysed with PlasmidFinder (Fig. 4b). Additionally, WGS results were further analysed in order to reconstruct the transferred plasmid that conferred ampicillin resistance in transconjugants (Fig. 4c).

While the ESBL *E. *coli donor harboured elements of 7 different plasmids, the recipients carried either no plasmids (*S.* London and *S.* Infantis), or 2 different IncF plasmids [IncFIB(S) and IncFII(S)] in case of *S.* Enteriditis, which were not found in the donor (Fig. 4a). The IncI1_1_Alpha plasmid was the only donor plasmid which was observed in all transconjugants assessed. Elements of the donor derived Col8282 and ColpVC plasmid elements were observed in some transconjugants, but not in the corresponding rif^R^ recipient (Fig. 4a). When looking at the resistance profiles of all strains, it can be seen that all strains harboured the *mdf*(A) gene which encodes a multidrug efflux pump (Fig. 4b)^35^. The aminoglycoside resistance gene *aac(6′)-Iaa* was only observed in recipient and transconjugant strains^36^. For the donor strain 11 different resistance genes were identified, of which two encoded beta-lactamases (*bla*_TEM-1B_ and *bla*_SHV-12_). Of these two beta-lactamase resistance genes only *bla*_TEM-1B_ was found in all transconjugants. These results indicated that the plasmid IncI1_1_Alpha, harbouring the *bla*_TEM-1B_ beta-lactamase resistance gene, was transferred from the donor to rif^R^ recipient strains during conjugation. This assumption was confirmed after WGS data was used to reconstruct IncI1_1_Alpha (Fig. 4c).

## Discussion

In this study, the IncI1_1_Alpha plasmid which contained the β-lactamase encoding gene *bla*_TEM-1B_ was successfully transferred from a donor *E. coli* strain to all three *Salmonella* recipient strains. This β-lactamase has been described previously in several studies analysing *Enterobacteriaceae* isolates of human and animal origin^37–39^. TEM-1 β-lactamases are known to be one of the most common plasmid-encoded β-lactamases in *Enterobacteriaceae*, mainly found on *Escherichia* and *Klebsiella* plasmids^40,41^. As described in a review of Smet et al.^42^, TEM-1 β-lactamases are not ESBLs as they can inactivate penicillins such as ampicillin but are unable to confer cephalosporin resistance. The *bla*_TEM-1_ β-lactamases have been found on plasmids of the incompatibility groups IncB/O, IncL/M, IncHI2/IncP, and IncF, but no known association between IncI and TEM-1 β-lactamases has been reported to date^43^.

One of the most interesting observations of this study was that conjugation frequencies and transconjugant numbers for the three tested *Salmonella* recipients differed from each other, with *S.* Infantis and *S.* London showing similar values, while *S.* Enteriditis showed lower conjugation frequencies in the conjugation assay. Notably, *S.* Enteriditis was the only recipient strain that harboured two additional IncF plasmids [IncFIB(S)_1 and IncFII(S)_1], thus plasmid incompatibility could have resulted in lower conjugation frequencies, as has been described previously^44,45^. However, in this study the IncF plasmids did not share the same replicon protein as the transferred plasmids, thus it is possible that other factors such as fitness costs associated with plasmid acquisition and carriage contributed to the observed differences in resistance and conjugation frequencies^46^.

When comparing growth of transconjugants with their parent recipient strains, *S.* London and *S.* Infantis were not impacted by carrying the transferred plasmid, while *S.* Enteriditis transconjugants showed an increased lag phase. As *S.* Enteriditis transconjugants carried evidence of elements from two other plasmids as well as the transferred plasmid, it is possible that carriage of multiple plasmids is associated with a fitness burden. No changes in the maximum growth rates were observed. During the lag phase, bacterial metabolic activity is dedicated to the adaption to a new environment, including potential stressors, nutrient acquisition in preparation for growth and cell division, whereas the exponential phase is mainly associated with processes involved in cell division^47^. Therefore, expression of genes of the newly acquired IncI1_1_Alpha plasmid such as *bla*_TEM-1B_ could be a greater burden during the lag phase than during exponential growth, which may explain why correlation between lag phase and maximum growth rates was not observed. Nevertheless, the length of the lag phase alone can be an indicator for cellular stress as described by Hamill et al.^47^ and could be associated with the differences in ampicillin resistance observed for the transconjugants. Furthermore, cellular stress associated with carriage of the IncI1_1_Alpha plasmid may also explain the lower conjugation frequencies of *S.* Enteriditis in comparison to the other recipient strains.

In the first conjugation assay, the effect of varying sub-inhibitory concentrations of Zn(NO_3_)_2_ and selective agents for the donor (rifampicin) and recipient strains (ampicillin) on conjugation frequencies was examined. While ampicillin decreased the conjugation frequencies for *S.* Infantis and *S.* London, rifampicin increased frequencies for these strains, although not significantly. Previous studies have shown that sub-inhibitory or sub-lethal selective pressure by antibiotics such as ciprofloxacin and amikacin can increase conjugation frequencies^8,11^. A possible explanation for these observations was given by Beaber et al.^9^. Exposure to antibiotics, independent of their class, induce intracellular stress, resulting in a SOS response and the expression of proteins involved in DNA recombination and repair, such as RecA. The protein RecA may then mediate the cleavage of repressor proteins such as LexA or SetR, allowing the transcription of genetic elements on the plasmid involved in the process^9,11,48^. Therefore, pressure exerted on the donor by rifampicin may promote conjugation frequencies. This effect was not observed for *S.* Enteriditis, where decreased conjugation frequencies were observed in the presence of rifampicin. However, two recent studies by Liu et al.^12^ and Shun-Mei et al.^49^ reported that conjugation frequencies do not always correlate with the SOS response and can be affected by SOS-independent mechanisms.

On the contrary, ampicillin induced selective pressure reduced conjugation frequencies for *S.* Infantis and *S.* London, while *S.* Enteriditis was unaffected. Beta-lactams such as ampicillin inhibit cell wall synthesis and therefore only affect growing cells. A study by Lee et al.^50^ showed a correlation between growth rates and efficacy of β-lactams. However, no significant differences in the maximum growth rates of the recipient strains were observed in this study. The results of the present study are in contrast to the findings of Liu et al.^12^, who observed that cefotaxime and ampicillin exposure increased conjugative transfer rates of an IncI1 plasmid, harbouring a *bla*_CTX-M-1_ gene, between ESBL *E. coli* donors and *E. coli* recipients. The reason behind the observed decrease in conjugation frequencies during exposure to ampicillin may indicate SOS-independent mechanisms, or strain variations, which remain elusive.

The presence of Zn(NO_3_)_2_ only affected conjugation frequencies with *S.* London. Interestingly, low concentrations of zinc (0.25 mM) resulted in a decrease in conjugation frequency in comparison to the control, while higher concentrations (1 mM) resulted in non-significant increases compared to the control (0 mM) but significant differences in comparison to the 0.25 mM assay. Buberg et al.^10^ reported that ZnCl_2_ reduced conjugative transfer rates of an IncK (more than 98%) and an IncI1 plasmid (more than 90%) in a concentration-independent but also concentration-dependent manner, with higher reduction at higher ZnCl_2_ concentration, respectively. Both plasmids in their study harboured the *bla*_CMY-2_ gene which encodes a plasmid-mediated AmpC-β-lactamase commonly found in *E. coli*^51^. Based on their transcriptional analysis via quantitative PCR, ZnCl_2_ significantly reduced the expression of *nikB* (encodes a conjugal transfer relaxase protein) and *traB* (encodes a Type IV secretion/conjugal transfer ATPase) which are both involved in the conjugation process. Consequently, Buberg et al.^10^ concluded that ZnCl_2_ reduces conjugation frequencies by interfering with conjugation associated processes and gene expression. Similar observations were made in a recent study by Crane et al.^11^ which focused on the effects of zinc and ciprofloxacin on conjugative transfer between an ESBL *Enterobacter cloacae* and an *E. coli* recipient*.* Although the plasmid type was not mentioned in their study, they reported that *bla*_CTX-M27_ was successfully transferred from donor to recipient. According to their study, ciprofloxacin increased conjugation frequencies by inducing the SOS response, while in combination with zinc acetate conjugation frequencies were reduced in a concentration-dependent manner by inhibiting the RecA-ssDNA binding as part of the SOS response. Thus, RecA-ssDNA binding may play an important role in the acquisition of transferred DNA in recipient cells^11^. Conversely, Zhang et al.^13^ reported that ZnSO_4_ increases conjugation frequencies, in a concentration-dependent manner. Their study investigated the transfer of the pCM184-Cm plasmid between *E. coli* strains, which carried multiple ARGs not further specified in their study. However, based on their results zinc had the lowest impact of all four tested heavy metals on HGT. Furthermore, the authors suggested multiple aspects that play a role in increased conjugation frequencies due to sub-inhibitory exposure to heavy metals: (1) production of intracellular reactive oxygen species and induction of the SOS response, (2) increased expression of *ompA* and *ompC*, which increased cell adhesion and thereby facilitated cell interactions^13,52^, (3) downregulation of global regulatory genes and upregulation of genes involved in HGT, such as genes encoding for relaxosomes. Whilst these studies show contradicting results, similar to the present study they all observe concentration dependency. It should also be noted that comparisons between studies are limited by the use of different strains, plasmids and different forms of zinc.

To further analyse the impact of time, conjugation was monitored by repeated sampling over time in a conjugation assay with *S.* London. The first transconjugants were observed after 4 h. Interestingly, the presence of zinc reduced conjugation frequencies at this time point although the observed reductions were not significant. However, differences in conjugation frequencies reduced with every measured time point until the same pattern as the original conjugation assay was observed after 24 h. Nevertheless, in contrast to the original conjugation assay the increase of conjugation frequency in the presence of 1 mM zinc was not significant. Notably, Zhang, et al.^13^, Buberg, et al.^10^, and Crane, et al.^11^ incubated donor and recipient strains together for different time periods: 4 h, 4 and 24 h, and 20 h respectively. The results of the present study clearly show that mating time plays an important role in conjugation experiments, as also discussed by Lopatkin, et al.^2^. Furthermore, as the donor and recipient cell populations increased during the 24 h conjugation assay, zinc concentrations per bacterial cell changed over time. Nevertheless, the growth of donor and recipient strains were not affected by the presence of zinc.

Concluding from these results, antimicrobials and heavy metals can affect AMR transfer in a strain- and concentration-dependent manner. Administration of antimicrobials and heavy metals on farms, may not only affect HGT events within the animal gut microbiota but also in the farm environment, considering that the farm environment may be contaminated with faeces, which can contain antimicrobial and heavy metal residues, posing a potential public health risk^53,54^. However, further investigations on the impact of antimicrobials and heavy metals on HGT events in more real world scenarios are necessary.

## Conclusion

In this study, the *bla*_TEM-1B_ resistance gene which conferred ampicillin resistance was successfully transferred from an ESBL *E. coli* donor strain to three different *S. enterica* serovars. Interestingly, conjugation frequencies varied between recipient strains, independent of exposure to antibiotics or zinc nitrate. While rifampicin exposure resulted in increased conjugation frequencies for two of the three recipient strains, ampicillin resulted in decreases of conjugation frequencies dependent on the concentration of the tested antibiotic. Zinc nitrate only affected one of the recipient strains, namely *S.* London, also in a concentration-dependent manner with the highest conjugation frequency observed at the highest zinc nitrate concentration. Transfer of the same plasmid into different recipient strains was shown to result in differing resultant MIC values for ampicillin, as well as a strain dependent impact on growth parameters. This study clearly demonstrates the importance of examining wider strain sets to determine the true biological meaning of observations in in vitro studies. Although several studies have focused on correlation between stress responses and conjugation frequencies using in vitro set-ups with individual strain pairs, future studies should also focus on additional factors that may influence HGT, as well as the expression and maintenance of acquired ARGs, to capture the complexity and influences on AMR spread via conjugative transfer in real world environments.

## Data Availability

All raw sequence data used for whole genome sequence analysis in this study is publicly available at the NCBI Sequence Read Archive (SRA) under the BioProject number PRJNA870297.
